# Symptoms and correlates of depression and anxiety in children and adolescents with juvenile idiopathic arthritis

**DOI:** 10.1186/s13023-026-04257-x

**Published:** 2026-02-18

**Authors:** Napapas Yothakol, Maynart Sukharomana, Sasitorn Chantaratin, Sirirat Charuvanij

**Affiliations:** 1 Division of Rheumatology, Department of Pediatrics, Faculty of Medicine Siriraj Hospital, Mahidol University, 2 Wanglang Road, Bangkok Noi, Bangkok, 10700 Thailand; 2 Division of Child and Adolescent Psychiatry, Department of Pediatrics, Faculty of Medicine Siriraj Hospital, Mahidol University, Bangkok, Thailand

**Keywords:** Anxiety, Children, Depression, Juvenile idiopathic arthritis, Mood, Mental, Psychological

## Abstract

**Objective:**

Juvenile idiopathic arthritis (JIA) can impair physical functioning and increase psychological burden. This study aimed to examine factors associated with clinically relevant depression and anxiety symptoms, and to describe the prevalence of clinically relevant depression and anxiety symptoms in children and adolescents with JIA.

**Methods:**

We conducted a prospective cross-sectional study from July 2020 to June 2022 among JIA patients aged 8‒17 years at the largest university-based tertiary hospital in Bangkok, Thailand. Clinically relevant depression and anxiety symptoms were measured using the Children’s Depression Inventory (CDI) and the Screen for Child Anxiety Related Disorders (SCARED).

**Results:**

Eighty-seven patients (50 males, 37 females) with a median age of 12.6 (IQR 9.8‒14.4) years were enrolled. The median disease duration was 38 (IQR 10‒67) months, and 61 (70.1%) exhibited clinically inactive disease. Nine (10.3%) had clinically relevant depressive symptoms, while 45 (51.7%) had clinically relevant anxiety symptoms. Among these 45, 34 (75.6%) exhibited separation anxiety, 16 (35.6%) social anxiety, 15 (33.3%) school anxiety, 6 (13.3%) panic symptoms, and 6 (13.3%) generalized anxiety. CDI scores were significantly correlated with SCARED scores (*r* = 0.519, *P* < 0.001). Multiple linear regression revealed that biologic therapy was associated with clinically relevant depressive symptoms (standard β = 0.353, 95%CI 3.064–13.286, *P* = 0.002), whereas disability was associated with clinically relevant anxiety symptoms (standard β = 0.325, 95%CI 0.998-10.153, *P* = 0.018).

**Conclusions:**

Symptoms of depression and anxiety were common even in JIA patients with clinically inactive disease. Routine mental health screening should be integrated into clinical care.

## Introduction

Juvenile idiopathic arthritis (JIA) is a chronic inflammatory joint disease of unknown etiology that affects children, leading to significant morbidity [1, 2]. JIA is characterized by onset of arthritis before 16 years of age that persists for at least 6 weeks and classified into 7 subtypes based on clinical presentations and number of joint involvement [1]. Globally, it impacts approximately 2 million children, presenting a range of clinical phenotypes [3]. In Thailand, systemic JIA and enthesitis-related arthritis are predominant, often resulting in substantial disability and complications such as joint contracture and leg-length discrepancy [4–6]. Although physical disability is well documented and plays a major role in reduced quality of life [7], the psychological effects stemming from the chronic, unpredictable course of JIA are also of great concern [8].

Adolescents with chronic physical conditions are particularly vulnerable to mental health issues, including depression and anxiety [9]. Studies have shown that children and adolescents with JIA exhibit higher rates of these psychological issues than their healthy peers [10–14]. For instance, female adolescents with JIA are more susceptible to mood and neurotic disorders [14]. Screening adolescents and young adults with JIA revealed a mental health burden of up to 32.7% [15]. Prior research indicates that depressive symptoms occur in 7%‒36% of patients with JIA, while anxiety symptoms range from 7% to 64% [16]. These internalizing symptoms may adversely affect disease management. Depression and anxiety can reduce motivation and treatment adherence, amplify pain perception, exacerbate functional limitations, and interfere with daily activities, including school performance and social interactions. These psychological comorbidities may impair health-related quality of life even more than JIA disease status itself [16].

Given the significant psychological burden in children and adolescents with JIA, early detection through mental health screening and prompt intervention is essential. However, data in Asian populations remain limited, underscoring the need for region-specific studies. Therefore, this study aimed to identify factors associated with clinically relevant depressive and anxiety symptoms and to describe the prevalence of these symptoms in children and adolescents with JIA in a Southeast Asian population.

## Materials and methods

### Study population

This prospective cross-sectional study included patients with JIA, aged 8‒17 years, who attended the pediatric rheumatology clinic at the Faculty of Medicine Siriraj Hospital, Mahidol University, Bangkok, Thailand, between July 2020 and June 2022. All patients were classified according to the International League of Associations for Rheumatology criteria [1]. Individuals unable to read and write or those with a preexisting psychiatric diagnosis were excluded.

### Data collection

Demographic, clinical, and treatment information was recorded, including age, sex, age of disease onset, disease duration, JIA subtype, clinical features, laboratory profiles, and medications received over the preceding 6 months. Disease activity was evaluated using the clinical Juvenile Arthritis Disease Activity Score (cJADAS) appropriate for each JIA subtype [17]. Inactive disease was defined according to the American College of Rheumatology provisional criteria, which require the absence of active arthritis, fever, rash, serositis, splenomegaly, or generalized lymphadenopathy attributable to JIA, as well as the absence of active uveitis, a normal erythrocyte sedimentation rate or C-reactive protein level, a physician global assessment at the best possible score, and morning stiffness lasting ≤ 15 min [18]. Functional disability was assessed using the Thai version of the Childhood Health Assessment Questionnaire (CHAQ) [19]. Moderate to severe disability was defined as a CHAQ score > 0.6 [6]. Data were obtained during the outpatient clinic visit. Clinical assessments were performed by pediatric rheumatologists.

Written informed consent was obtained from parents, and assent was obtained from patients. The Siriraj Institutional Review Board approved this study (COA 191/2020).

### Mental health questionnaires

The Children’s Depression Inventory (CDI) Thai version [20] and the Screen for Child Anxiety Related Disorders (SCARED) Thai version [21] were provided to all participants. The CDI is a 27-item self-report instrument with total scores ranging from 0 to 54 [22]. A cutoff score of > 15 indicates clinically relevant depressive symptoms, with 79% sensitivity and 91% specificity for depression in Thai children [20].

Anxiety symptoms were assessed using the 41-item Thai version of the SCARED, which yields total scores ranging from 0 to 82 [21]. This questionnaire evaluates anxiety across five subdomains: separation anxiety disorder, generalized anxiety, social phobia, school phobia, and panic symptoms [23]. Clinically relevant anxiety symptoms were defined as a total SCARED score ≥ 25 or the presence of scores meeting the established cutoff in at least one of the SCARED subscales [23]. Data were obtained during the same outpatient clinic visit.

### Statistical analysis

All data were analyzed using IBM SPSS Statistics, version 22 (IBM Corp, Armonk, NY, USA). Descriptive data are presented as frequencies and percentages for categorical variables, and as mean with standard deviation or median (IQR) for continuous variables, as appropriate. Group comparisons were performed using the chi-square or Fisher’s exact test for categorical variables, and the Mann‒Whitney *U* or Kruskal‒Wallis test for continuous variables. Pearson’s correlation coefficient (*r*) or Spearman’s correlation (ρ) was applied, as appropriate, to evaluate correlations between variables. Multiple linear regression with the enter method was used to identify factors associated with CDI and SCARED. The independent variables were selected based on bivariate significance by selecting significant variables from the univariable analysis. Additionally, the variables “JIA subtype”, “cJADAS”, “age” and “sex” were added in the final model to adjust for the potential confounders of JIA disease subtype and disease activity. Statistical significance was set at *P* < 0.05.

## Results

### Participant characteristics

This cross-sectional analysis was conducted between July 2020 and June 2022. All consecutive patients with JIA diagnosis who attended the pediatric rheumatology clinic during this period were approached for inclusion. After applying the eligibility criteria, a total of 87 patients were included in the final analysis. No participants were excluded from the study.

Among 87 participants, 50 (57.5%) were male and 37 (42.5%) were female. Their median age at enrollment was 12.6 years (IQR 9.8‒14.4). The JIA subtypes were as follows: enthesitis-related arthritis in 34 patients (39.1%), systemic JIA in 24 (27.6%), oligoarticular JIA in 9 (10.3%), polyarticular JIA rheumatoid factor negative in 8 (9.2%), polyarticular JIA rheumatoid factor positive in 8 (9.2%), and undifferentiated JIA in 4 (4.6%). The median follow-up time was 38 months (IQR 10‒67), and the median cJADAS was 0 (IQR 0‒4). Sixty-one patients (70.1%) had inactive disease. Moderate to severe disability was observed in 22 patients (25.3%), and the median Visual Analog Scale (VAS) pain score was 0 (IQR 0–10). Median erythrocyte sedimentation rate was 14 mm/h (IQR 8‒23), while the median C-reactive protein level was 1.1 mg/L (IQR 0.3‒5.9).

Regarding medications, 64 patients (73.6%) were receiving treatment for JIA. The total number of medications ranged from 0 to 3, with a median of 1. Nonsteroidal anti-inflammatory drugs (NSAIDs) were used by 44 patients (50.6%), prednisolone by 24 (27.6%), and methotrexate by 54 (62%). Biologic therapy was prescribed for 6 patients (6.8%), and subcutaneous injections were administered in 9 (10.3%). Detailed demographic and clinical characteristics are presented in Table 1.

**Table 1:** Demographic and clinical characteristics of patients with juvenile idiopathic arthritis (*N* = 87)

Characteristics	*n* (%), median (IQR)
Male, n (%)	50 (57.5)
Female, n (%)	37 (42.5)
Age at study visit (y)	12.6 (9.8-14.4)
Subtypes	
Enthesitis-related arthritis	34 (39.1)
Systemic	24 (27.6)
Oligoarthritis	9 (10.3)
Polyarticular, RF–	8 (9.2)
Polyarticular, RF+	8 (9.2)
Undifferentiated	4 (4.6)
Disease duration, median (IQR), months	38 (10-67)
Clinical features	
Arthritis	19 (21.8)
Enthesitis	5 (5.7)
Uveitis	4 (4.6)
JIA variables	
Number of active arthritis	0 (0-8)
Number of enthesitis	0 (0-2)
Number of limited ROM	0 (0-1)
PGA	0 (0-2)
PGW	0 (0-2)
CHAQ	0 (0-0.5)
ESR, mm/h	14 (8-23)
CRP, mg/dL	1.1 (0.3-5.9)
JIA disease status	
Active	26 (29.9)
Inactive	61 (70.1)
cJADAS	0 (0-4)
Moderate to severe disability	22 (25.3)
Treatment	
NSAID	44 (50.6)
Prednisolone	24 (27.6)
Methotrexate	54 (62)
Sulfasalazine	7 (8)
Cyclosporin A	2 (2.2)
Etanercept	3 (3.4)
Tocilizumab	3 (3.4)
Number of medications	1 (0-3)
Subcutaneous injection	9 (10.3)

Abbreviations: CHAQ, Childhood Health Assessment Questionnaire; cJADAS, clinical Juvenile Arthritis Disease Activity Score; CRP, C-reactive protein; ESR, erythrocyte sedimentation rate; JIA, juvenile idiopathic arthritis; NSAID, non-steroidal anti-inflammatory drug; PGA, Physician Global Assessment of Activity; PGW, Patient/Parents Global Assessment of Well-Being; RF, rheumatoid factor; ROM, range of motion. Values are presented as n (%), median (IQR), or as specified

### Mental health outcomes

Clinically relevant depressive symptoms were observed in 9 patients (10.3%), of whom 6 (66.7%) were female. The median CDI score in this group was 20 (IQR 17.0‒22.0). Clinically relevant anxiety symptoms were documented in 45 patients (51.7%), with separation anxiety being the most common subscale (34 patients, 75.6%). Social anxiety affected 16 (35.6%), school anxiety 15 (33.3%), panic symptoms 6 (13.3%), and generalized anxiety 6 (13.3%), as shown in Fig. 1A. The number of anxiety subscales reported by these patients is illustrated in Fig. 1B. The median total SCARED score in the group with clinically relevant anxiety symptoms was 22 (IQR 18.0‒28.5).

**Fig. 1A:**
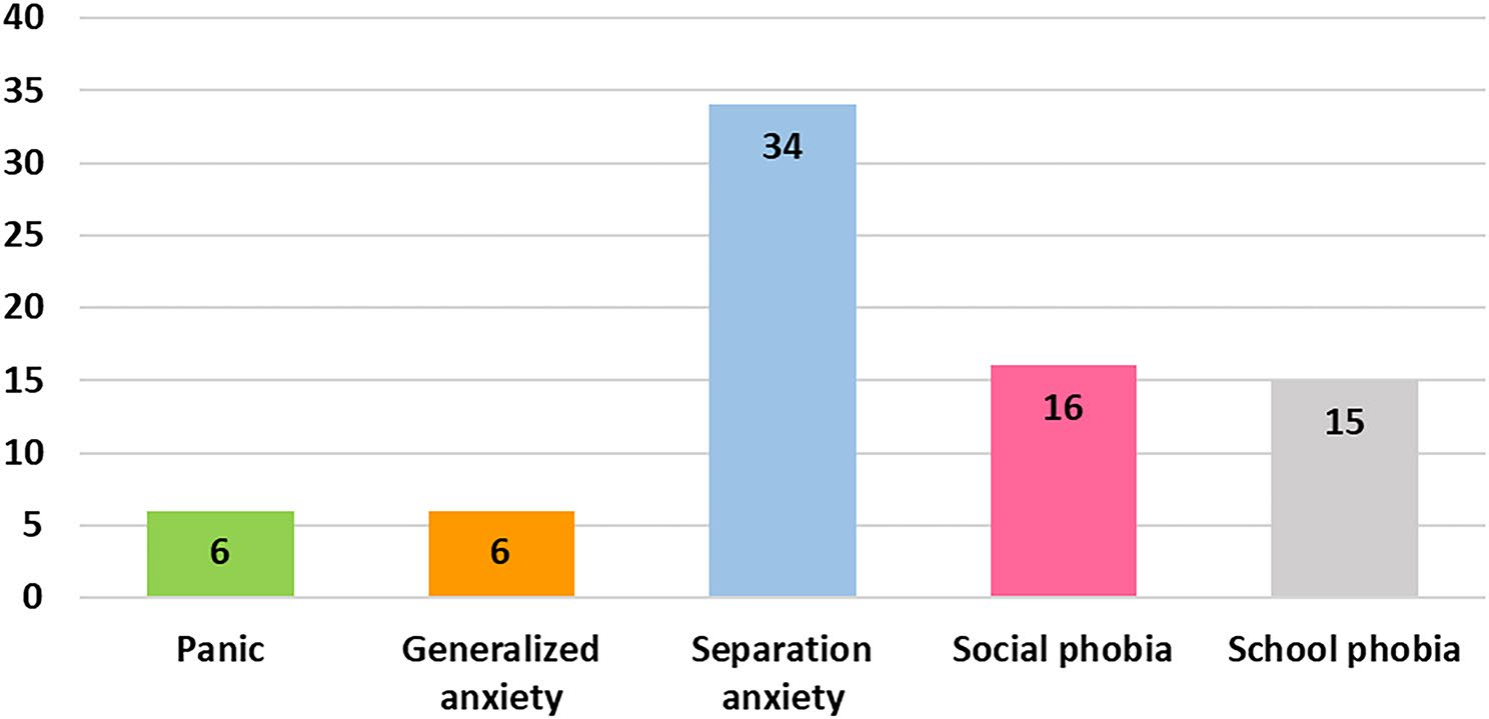
Categories of clinically relevant anxiety symptoms in children and adolescents with juvenile idiopathic arthritis (*n* = 45)

**Fig. 1B:**
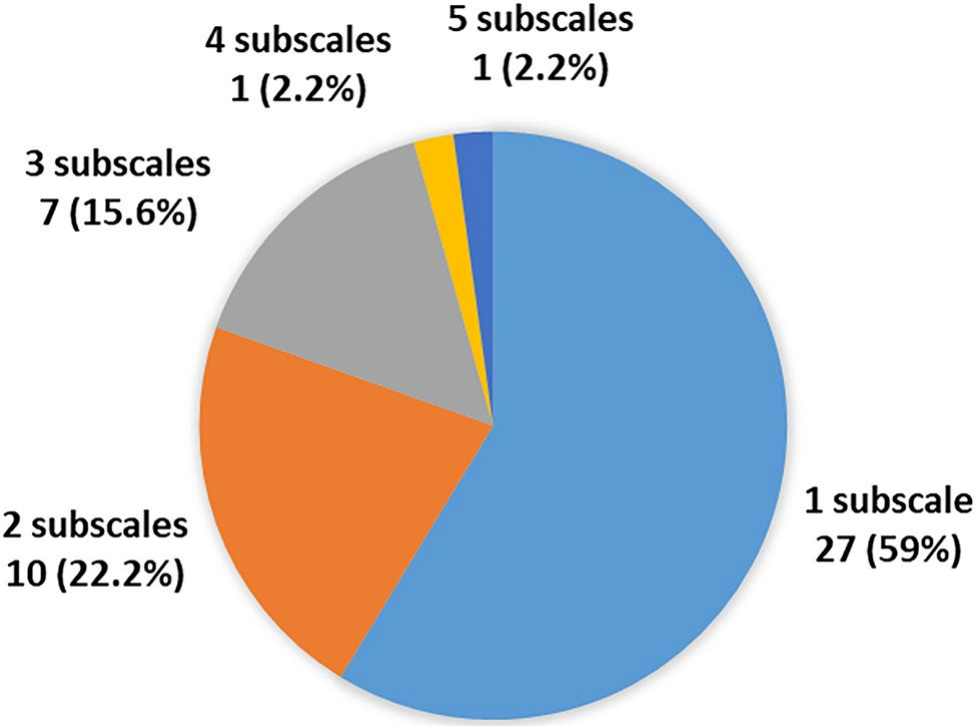
Number of clinically relevant anxiety symptom subscales reported by juvenile idiopathic arthritis patients (*n* = 45)

Biologics and subcutaneous injections were more common among participants with clinically relevant depressive symptoms (CDI > 15). Patients who received biologics exhibited clinically relevant depressive symptoms (*P* = 0.001). Patients who received subcutaneous injections revealed marginally clinically relevant depressive symptoms (*P* = 0.048). However, disease duration, moderate to severe disability, JIA disease activity, pain, and cJADAS were not significantly associated with clinically relevant depressive symptoms (Table 2). In contrast, clinically relevant anxiety symptoms were associated with higher CHAQ scores (*P* = 0.018), elevated VAS pain scores (*P* = 0.041), and a greater number of medications received (*P* = 0.042; Table 3).

**Table 2:** Comparison of patients with and without clinically relevant depressive symptoms

	With clinically relevant depressive symptoms (*n* = 9)	Without clinically relevant depressive symptoms (*n* = 78)	*P* value
CDI score	20 (17.0–22.0)	5 (2.0–9.0)	< 0.001*
Age (years), median (IQR)	9.9 (9.4–13.6)	12.6 (10.0-14.4)	0.231
Female (%)	6 (66.7%)	31 (39.7%)	0.161
Systemic JIA (%)	3 (33.3%)	21 (26.9%)	0.702
Duration of disease (months), median (IQR)	46 (27.5–74.0)	38 (8.7–66.2)	0.414
Disease duration > 12 months (%)	8 (88.9%)	53 (67.9%)	0.269
Clinical features			
Arthritis (%)	1 (11.1%)	18 (23.1%)	0.677
Enthesitis (%)	0	5 (6.4%)	1.000
Uveitis (%)	0	4 (5.1%)	1.000
JIA disease status			
Disease active (%)	2 (22.2%)	24 (30.8%)	0.719
cJADAS, median (IQR)	1 (0–6)	0 (0-4.25)	0.648
PGA (0–10), median (IQR)	0 (0–2)	0 (0–2)	0.709
PGW (0–10), median (IQR)	0 (0-3.5)	0 (0–2)	0.614
CHAQ score (0–3), median (IQR)	0.25 (0-1.13)	0 (0-0.38)	0.118
CHAQ > 0.6 (%)	4 (44.4%)	18 (23.1%)	0.222
VAS pain (0-100), median (IQR)	0 (0–55)	0 (0–10)	0.282
ESR (mm/h), median (IQR)	20.5 (6.2–39.7)	13 (8–23)	0.634
CRP (mg/dL), median (IQR)	0.9 (0.3–1.5)	1.12 (0.3–6.5)	0.541
Treatment			
Receiving medications (%)	8 (88.9%)	56 (71.8%)	0.435
NSAID (%)	3 (33.3%)	41 (52.6%)	0.314
Methotrexate (%)	4 (44.4%)	44 (56.4%)	0.507
Sulfasalazine (%)	1 (11.1%)	6 (7.7%)	0.548
Cyclosporin (%)	1 (11.1%)	1 (1.3%)	0.197
Biologics (%)	4 (44.4%)	2 (2.6%)	0.001*
Subcutaneous injection (%)	3 (33.3%)	6 (7.7%)	0.048*
Medication amount, median (IQR)	2 (1–3)	1 (0–3)	0.282

Abbreviations: CDI, Children’s Depressive Inventory; CHAQ, Childhood Health Assessment Questionnaire; cJADAS, clinical Juvenile Arthritis Disease Activity Score; CRP, C-reactive protein; ESR, erythrocyte sedimentation rate; JIA, juvenile idiopathic arthritis; NSAID, non-steroidal anti-inflammatory drug; PGA, Physician Global Assessment of Activity; PGW, Patient/Parents Global Assessment of Well-Being; VAS, visual analogue scale

Values are expressed as n (%), median (IQR), or as indicated

* *P* < 0.05 is considered statistically significant

**Table 3:** Comparison of patients with and without clinically relevant anxiety symptoms

	With clinically relevant anxiety symptoms (*n* = 45)	Without clinically relevant anxiety symptoms (*n* = 42)	*P* value
SCARED score	22 (18.0-28.5)	11 (8.5–15.0)	< 0.001*
Age (years), median (IQR)	11 (9.7–14.1)	13 (10.9–14.8)	0.177
Female (%)	23 (51.1%)	14 (33.3%)	0.094
Systemic JIA (%)	14 (31.1%)	10 (23.8%)	0.446
Duration of disease (month), median (IQR)	37 (6.0-64.5)	41.5 (15.7–69.0)	0.390
Disease duration > 12 months (%)	28 (62.2%)	33 (78.6%)	0.096
Clinical features			
Arthritis (%)	11 (24.4%)	8 (19.0%)	0.543
Enthesitis (%)	2 (4.4%)	3 (7.1%)	0.669
Uveitis (%)	2 (4.4%)	2 (4.8%)	0.944
JIA disease status			
Disease active (%)	15 (33.3%)	11 (26.2%)	0.467
cJADAS, median (IQR)	1 (0-6.50)	0 (0-2.50)	0.144
PGA (0–10), median (IQR)	0 (0–2)	0 (0–1)	0.210
PGW (0–10), median (IQR)	0 (0-2.5)	0 (0–1)	0.109
CHAQ score (0–3), median (IQR)	0.13 (0-0.94)	0 (0-0.28)	0.018*
CHAQ > 0.6 (%)	14 (31.1%)	8 (19.0%)	0.196
VAS pain (0-100), median (IQR)	0 (0–20)	0 (0-1.25)	0.041*
ESR (mm/h), median (IQR)	14 (9–30)	10.5 (6.0-21.2)	0.082
CRP (mg/dL), median (IQR)	0.6 (0.3–9.2)	2.3 (0.4–5.6)	0.280
Treatment			
Receiving medications (%)	36 (80.0%)	28 (66.7%)	0.159
NSAID (%)	25 (55.6%)	19 (45.2%)	0.336
Methotrexate (%)	27 (60.0%)	21 (50.0%)	0.349
Sulfasalazine (%)	5 (11.1%)	2 (4.8%)	0.435
Cyclosporine (%)	2 (4.4%)	0	0.495
Biologics (%)	4 (8.9%)	2 (4.8%)	0.677
Subcutaneous injection (%) Medication amount, median (IQR)	4 (8.9%) 2 (1–3)	5 (11.9%) 1 (0-2.2)	0.733 0.042*

Abbreviations: CHAQ, Childhood Health Assessment Questionnaire; cJADAS, clinical Juvenile Arthritis Disease Activity Score; CRP, C-reactive protein; ESR, erythrocyte sedimentation rate; JIA, juvenile idiopathic arthritis; NSAID, non-steroidal anti-inflammatory drug; PGA, Physician Global Assessment of Activity; PGW, Patient/Parents Global Assessment of Well-Being; SCARED, screen for Child Anxiety Related Disorders; VAS, visual analogue scale

Values are expressed as n (%), median (IQR), or as indicated

* *P* < 0.05 is considered statistically significant

**Fig. 2:**
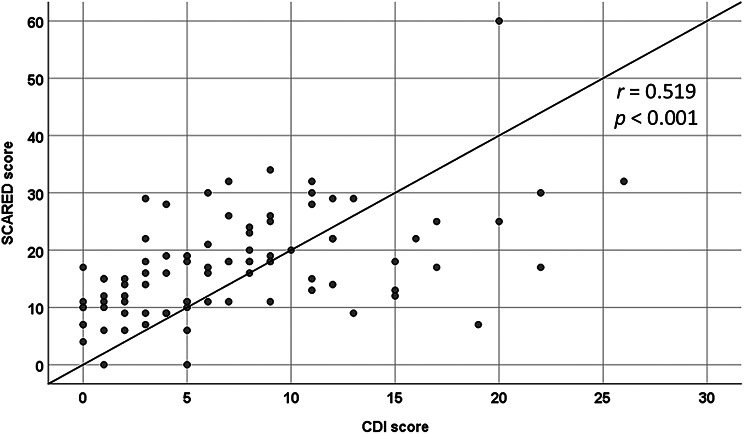
Correlation between children’s depression inventory scores and screen for child anxiety related disorders scores in patients with juvenile idiopathic arthritis

CDI scores were positively correlated with SCARED scores (*r* = 0.519, *P* < 0.001), as illustrated in Fig. 2. Both CDI and SCARED scores were also positively correlated with CHAQ (*r* = 0.217, *P* = 0.043 and *r* = 0.274, *P* = 0.010, respectively) and VAS pain (ρ = 0.244, *P* = 0.023 and ρ = 0.227, *P* = 0.034, respectively). Multiple linear regression analysis revealed that biologics treatment was associated with clinically relevant depressive symptoms (standard β = 0.353, 95%CI 3.064–13.286, *P* = 0.002), whereas disability correlated with clinically relevant anxiety symptoms (standard β = 0.325, 95%CI 0.998-10.153, *P* = 0.018; Table 4).

**Table 4:** Multiple linear regression analysis of factors associated with CDI score and SCARED score

Dependent variable	Independent variable	Standardized coefficient β	Unstandardized coefficient		95% CI for B	*P* value	R	R^2^
			Intercept (B)	SE				
CDI score	Biologics	0.353	8.175	2.568	3.064–13.286	0.002*	0.400	0.160
	Subtype of JIA	0.159	0.504	0.332	–0.156–1.165	0.132		
	cJADAS	0.084	0.110	0.139	–0.166 – 0.386	0.430		
	Subcutaneous injection Age Sex	–0.054 0.223 0.207	–1.033 0.153 2.461	2.166 0.073 1.249	–5.344–3.278 − 0.291-0.598 − 0.024-4.947	0.635 0.494 0.052		
SCARED score	CHAQ	0.325	5.576	2.300	0.998–10.153	0.018*	0.434	0.189
	Subtype of JIA	–0.151	–0.739	0.510	–1.754 – 0.276	0.151		
	cJADAS	–0.138	–0.276	0.311	–0.896– 0.343	0.377		
	VAS for pain	0.061	0.026	0.073	–0.119 – 0.172	0.720		
	Medication amount Age Sex	0.051 0.109 0.266	0.353 0.352 4.863	0.863 0.360 1.906	–1.364–2.070 − 0.365-1.069 1.069–8.658	0.684 0.332 0.013*		

Abbreviations: CDI, Children’s Depressive Inventory; CHAQ, Childhood Health Assessment Questionnaire; 95% CI, 95% confidence interval; cJADAS, clinical Juvenile Arthritis Disease Activity Score; JIA, juvenile idiopathic arthritis; SCARED, Screen for Child Anxiety Related Disorders; SE, standard error; VAS, visual analogue scale

* *P* < 0.05 is considered statistically significant

## Discussion

This study identified a substantial mental health burden among children and adolescents with JIA. More than half of our participants experienced psychological symptoms, with anxiety more prevalent than depression. Separation anxiety was the most common subscale, followed by social and school-related anxiety. Disability and the use of biologic therapy were associated with clinically relevant anxiety and depressive symptoms, respectively.

Managing chronic pain, disease-related damage, reduced functional ability, multiple medications, and prolonged treatment can impair quality of life for children and adolescents with JIA and increase their susceptibility to psychological problems [4, 6, 7]. Additionally, chronic joint inflammation can influence neurotransmitters and thereby affect psychological status [24]. Most patients in our study reported no pain evidenced by the VAS of 0 (IQR 0–10), likely reflecting adequate disease control with ongoing treatment. In patients with JIA, functional limitation often remains more prominent than pain, which helps explain why a subset of patients continued to experience moderate to severe disability despite minimal pain scores. Moreover, disability measures by the CHAQ assess broader domains of daily functioning, which may remain impaired even when pain is minimal.

In our study, 10.3% of participants had clinically relevant depressive symptoms, as measured by the CDI. This rate is noteworthy because most of our patients had inactive disease, yet some still experienced depressive symptoms. Similarly, a south Swedish JIA cohort reported a 14.5% prevalence of depression [25]. Roemer et al. described depressive symptoms in 13% of German children and adolescents with JIA, with 71.4% newly diagnosed [26]. The Childhood Arthritis Prospective Study in the United Kingdom found a prevalence of 14.7% [27]. Notably, Milatz et al. observed depressive symptoms in 25.8% of their sample, as measured by the Patient Health Questionnaire-9, and 12.2% reported suicidal or self-harm thoughts [15]. Abdul-Sattar et al. likewise reported that 30.7% of their JIA cohort showed depressive symptoms on the CDI, which correlated with school absenteeism and poor academic performance [28]. In addition, Kayan Ocakoglu et al. documented a 31.2% prevalence of mood disorders in a small group of JIA patients, primarily depressive disorders [29]. Prevalence estimates of depressive symptoms in children and adolescents with JIA vary across studies, partly reflecting methodological differences. Importantly, studies using formal psychiatric interviews are unable to directly comparable with those using self-report screening tools. Diversity across studies may also be influenced by each cohort characteristics including age, gender and JIA subtypes. Cultural stigma and underreporting may also play the role. Taken together, these differences highlight the need for careful interpretation when comparing prevalence rates of depressive symptoms across studies.

A relatively lower prevalence of depression in our study may arise from differences in assessment tools or cultural influences. Some individuals may underreport depressive symptoms due to the stigma surrounding mental health issues, and adolescents with JIA may possess psychological resilience or effective coping strategies that mitigate depressive symptoms [30]. Future research should explore the most accurate methods for detecting depression in this population. Notably, our study did not find an association between JIA disease activity, pain, or disability and depression, which contrasts with previous reports [27, 31, 32]. It should be mentioned that the majority of our cohort had inactive disease and low pain scores, which may have limited the variability needed to detect the association with clinically relevant depressive symptoms. Although a small number of patients with JIA received biologics, we observed that clinically relevant depressive symptoms were associated to the use of biologic therapies, possibly reflecting the emotional burdens of frequent hospitalizations for intravenous infusions or the self-perception of having a severe disease.

Regarding the relatively low rate of biologics use in our study, this finding reflects the real-world practices in the resource-limited settings. In Thailand, biologics were not universally accessible due to cost constraints and limited reimbursement policies, even within tertiary care centers [4, 6]. Combination of NSAID, conventional disease-modifying antirheumatic drugs (DMARDs) and/or corticosteroids remain the mainstay of therapy, and biologics are reserved for patients with refractory disease.

Clinically relevant Anxiety symptoms were notably frequent, affecting more than half of our participants. Previous studies have reported anxiety prevalence rates of 7%‒64% in children and adolescents with JIA [16]. For instance, one study found that JIA patients had higher anxiety and lower health-related quality of life across multiple subscales compared to healthy controls [33]. Similarly, children with JIA have been shown to exhibit higher rates of anxiety and somatic complaints [10]. In contrast, Berthold et al. did not observe an increased risk of anxiety (17.3%) among JIA patients compared to matched controls [25].

In contrast to the findings of Bomba et al., who reported social anxiety as the most common subtype [33], separation anxiety was most prevalent in our cohort. This difference may be partly explained by the younger average age of our participants, cultural factors such as closer parental involvement in Thai families, and the low overall disease activity in our sample, which may reduce exposure to peer-related social challenges. Additionally, variations in screening tools and cutoff criteria across studies may have contributed to this discrepancy.

Approximately one-third of patients with JIA in our study had school-related anxiety. School-related anxiety in JIA has also been noted, encompassing concerns about physical activity (42.1%), social interactions with peers (32.2%), and higher rates of class absences due to clinic appointments (70.1%) [34]. This underscores the importance of educational contexts in shaping the psychological well-being of children and adolescents with JIA within the school environment.

Our study further indicated that based on multiple linear regression analysis clinically relevant anxiety symptoms measured by the SCARED tool associated with functional disability measured by the CHAQ than with JIA disease severity, underscoring the importance of early interventions aimed at reducing disability-related stress. Addressing both psychological and functional challenges are pivotal for comprehensive JIA management. Previous research has shown that anxiety in JIA is significantly associated with disability, pain, and physician global assessment [31]. Although anxious preoccupation has been linked to active disease and increased disability in some studies [35], prior study has not demonstrated associations between anxiety and inflammatory markers such as interleukin-6 or C-reactive protein [31]. This distinction highlights that psychological distress in pediatric rheumatic diseases may not directly reflect inflammatory activity and stresses the importance of evaluating psychosocial outcomes independently of biological disease markers. Consistent with findings by Tarakci et al. [36], our results also demonstrated a correlation between depression and anxiety in JIA, which may be explained by the fact that both are internalized emotional disturbances.

Our findings should be interpreted with caution, given the study’s cross-sectional design which limits the ability to draw causal inferences. The relatively small sample size from a tertiary care center, in part due to limited clinic access during the COVID-19 pandemic [37], also restricts generalizability. The pandemic itself may have increased psychosocial stressors in children and adolescents with JIA, potentially contributing to clinically relevant anxiety or depressive symptoms in our study [37]. The limited sample size also affects the statistical power of our subgroup analyses and regression models, particularly for variables with small subsample counts such as biologics treatment. Given the very small number of patients receiving biologics, the observed association with clinically relevant depressive symptoms should be regarded as exploratory. Although some associations reached statistical significance, both regression models explained only a modest proportion of the variance in symptom scores. Moreover, the absence of a control group prevents direct comparisons with healthy populations. Therefore, it is not possible to determine whether the prevalence of clinically relevant depressive and anxiety symptoms observed in our cohort is specific to JIA or reflects a broader psychosocial burden among Thai children. The predominance of inactive JIA disease in this study likely influenced the observed lack of association between clinically relevant depression and anxiety symptoms and disease activity. Additionally, recruitment proceeded mainly with routine follow-up visits of most stable patients rather than acute presentations; therefore, the potential of selection bias should be acknowledged. It should also be noted that formal psychiatric evaluations are required to confirm psychiatric diagnoses.

Despite these limitations, our results underscore the value of psychological screening in Southeast Asian children and adolescents with JIA. Depression and anxiety symptoms were common, emphasizing the need for early detection. Routine psychological assessments should be incorporated into pediatric rheumatology care, and future research should further investigate the mental health and stress experienced by caregivers of JIA patients.

## Data Availability

The data that support the findings of this study are not publicly available due to privacy reasons but are available from the corresponding author upon reasonable request.

## Abbreviations

CDI: Children’s Depression Inventory
CHAQ: Childhood Health Assessment Questionnaire
CI: Confidence interval
CRP: C-reactive protein
ESR: Erythrocyte sedimentation rate
IQR: Interquartile range
JADAS: Juvenile Arthritis Disease Activity Score
JIA: Juvenile Idiopathic Arthritis
NSAID: Non-steroidal anti-inflammatory drug
SCARED: Screen for Child Anxiety Related Disorders
PGA: Physician Global Assessment of Activity
PGW: Patient/Parents Global Assessment of Well-Being
VAS: Visual analogue scale
